# The influence of therapy quality on outcomes from behavioural activation and guided self‐help treatments for depression in adults with intellectual disabilities

**DOI:** 10.1111/bjc.12502

**Published:** 2024-09-03

**Authors:** Dave Dagnan, Paul Thompson, Richard P Hastings, Chris Hatton, Chris Melville, Sally‐Ann Cooper, Nicola McMeekin, Lauren Fulton, Rob S. P. Jones, Alex McConnachie, Andrew Jahoda

**Affiliations:** ^1^ Community Learning Disability Services Cumbria, Northumberland, Tyne & Wear NHS Foundation Trust Workington UK; ^2^ University of Cumbria Carlisle UK; ^3^ Centre for Research in Intellectual and Developmental Disabilities University of Warwick Coventry UK; ^4^ Manchester Metropolitan University Manchester UK; ^5^ School of Mental Health and Wellbeing University of Glasgow Glasgow UK; ^6^ School of Psychology Bangor University Bangor Gwynedd UK

**Keywords:** behavioural activation, depression, guided self‐help, intellectual disability, therapist adherence, therapist competence, therapy quality

## Abstract

**Objectives:**

We report the effect of quality of therapy delivery on outcomes in a randomized, controlled trial of behavioural activation (BA) and guided self‐help (GSH) for depression in adults with intellectual disabilities.

**Methods:**

A study specific measure of quality was used in a linear mixed effect model to determine the effects therapy and therapy quality on therapy outcome.

**Results:**

There was a significant interaction between quality and treatment type, with lower quality therapy associated with better outcome for GSH but poorer outcome for BA, with little difference in outcomes at higher levels of therapy quality.

**Conclusions:**

Factors suggesting high quality in individualized BA may indicate problematic engagement for GSH. More research into processes in therapy for people with intellectual disabilities is required.

**Practitioner points**

- There is little research on the effect of the quality of therapy delivery on therapy outcome for therapy with people with intellectual disabilities.
- Factors indicating therapy quality in therapy with people with intellectual disabilities may be different for therapies with different therapist and client demands.

## INTRODUCTION

Common factors have been identified in therapy; these can include specific components such as ‘adherence and competence’ (Wampold, 2015). ‘Adherence’ is the delivery of specific therapy components expected in a defined therapy or manual and may include aspects of consistency with a therapy model; therapy ‘competence’ refers to the skill with which a therapist delivers the therapy (Power et al., 2022). Adherence and competence are associated and both may be required in the ideal delivery of evidence‐based therapy (Waltz et al., 1993). Studies linking Cognitive Behaviour Therapy (CBT) competence to client outcomes have not given consistent results, for example, Branson et al. (2015) did not find a linear effect of therapist competence on therapy outcome, but the top 10% of competent therapists had better outcomes and the bottom 10% of competent therapists had least good outcomes.

In intellectual disability research, three studies have reported the measurement of therapy adherence or competence. Two studies used variants of the Cognitive Therapy Scale (CTS; Vallis et al., 1986), a measure completed by observers that identifies components such as feedback, understanding, collaboration, focus on cognition and strategy for change. Hassiotis et al. (2013) and Jahoda et al. (2009) demonstrate that therapy with people with intellectual disabilities used recognizable cognitive behavioural techniques, an important consideration in using these interventions with this group.

Jahoda et al. (2013) reported adherence and competence for an anger management intervention delivered by social care staff to people with intellectual disabilities. They used a specific measure of adherence and competence for manualized, group‐based CBT interventions for people with intellectual disabilities. There were significant positive correlations reported for this measure with post‐intervention anger provocation responses (*r* = .43, *p* < .001) and anger skills (*r* = .26, *p* < .02), although this analysis did not account for baseline anger scores.

Jahoda et al. (2017) reported a multi‐centre, single‐blind, randomized controlled trial that compared adapted BA (BeatIt) to GSH (StepUp) with participants randomly assigned to interventions (84 to BeatIt; 77 to StepUp). Findings from the trial showed no group differences in primary outcome (Glasgow Depression Scale for people with a Learning Disability, GDS‐LD; Cuthill et al., 2003) at 12 months. However, both groups showed improvements in outcome indicating both treatments may be active and effective; neither intervention was ‘treatment as usual’ at the request of the funder. This study included measures of both adherence to study manuals and adherence to model and competence in delivery. Adherence to manual is reported in the main study (Jahoda et al., 2018), however, the impact of adherence to model and competence in delivery (hereon referred to as therapy ‘quality’) on therapy outcome has not been previously reported and the purpose of this study is to evaluate whether this moderates treatment effect.

## METHODS

### Participants

Participant recruitment processes are reported in the main study paper and 93 out of 161 (58%) participants had therapy quality data from recordings of sessions (Jahoda et al., 2018). Missing therapy quality data was due to 21 participants who refused consent for recording, 39 participants who withdrew from the study before being recorded, 12 participant or carers who refused to be recorded on the day and two recordings that were lost in transfer or as a result of other technical difficulties with recording equipment. No differences in participant (baseline scores, other covariates, age or sex) or therapist characteristics (profession, sex and years of experience) were found between those with and without therapy quality information. No significant differences in therapy quality were found between the two intervention groups (32.3 [*SD* 4.1] for BeatIt, 33.9 [*SD* 3.9] for StepUp).

West of Scotland Research Ethics Committee 3 gave approval for the study (NRES: 13/MH97). The approval included agreement for moderator analysis with a range of baseline and process variables. The full trial protocol has been published (Jahoda et al., 2015).

### Measures and variables

Therapy Quality Measure–Intellectual Disabilities (TQM‐ID; Jahoda et al., 2018): a 10‐item quality measure for structured, manualized and individualized therapies. The measure was based on scales described by Hunter et al. (2012) which were, themselves based on the CTS‐R (Blackburn et al., 2001). The full description and development of the TQM‐ID is reported in Jahoda et al. (2018); the scale includes items such as whether the therapist maintains a focus and clear structure to the session; asks for feedback and reaction to the current session; adjusts content and style of own communication; shows empathy; and shows warmth and respect.

The TQM‐ID was scored from recorded sessions, with the recorded sessions assigned by the researchers to ensure coverage across therapy sessions. Each of the items were rated on a 1–4 scale with a higher score indicating better quality. Jahoda et al. (2017) reported that the scale has an alpha of .76 and a mean‐adjusted item‐total correlation of .42 (*SD*: .10, range: .31–.58). The methodology for establishing and maintaining reliability of coding is reported fully in Jahoda et al. (2018).

#### Outcome measure

Both the Glasgow Depression (GDS‐LD; Cuthill et al., 2003) and Glasgow Anxiety (GAS‐ID; Mindham & Espie, 2003) Scales were available; the GDS‐LD was the primary outcome, and both the GDS‐LD and GAS‐ID were used as baseline measures in the analyses.

This study took place in three geographical areas, the potential impact of supervision and clinical leadership in these areas was accounted for by including site as a fixed effect as was time point of quality assessment to test whether any between‐group differences varied over time (Cameron et al., 2018).

### Statistical analysis

The primary analysis, from the original study, compared GDS‐LD scores at 4 and 12 months separately after randomization between intervention groups; this study also reported analysis that included both 4‐ and 12‐month time points in the same analysis and all further analyses in this paper will be with reference to this model. Thus, a linear mixed‐effects regression model was used with adjustment for baseline GDS‐LD scores, study centre and time point as fixed effects and random intercepts for therapists and participants.

To estimate the influence of therapy quality on the intervention, we use a moderator analysis with a fixed effect of therapy quality and include the interaction between TQM‐ID score and a binary dummy variable for intervention. The TQM‐ID score was grand mean centred to improve interpretability. Two additional covariates included that impact on therapy quality and outcome were the session number at which recording was made (Cameron et al., 2018) and baseline anxiety scores (Melville et al., 2023).

In the original trial, analyses were repeated with multiple imputation; this checked findings' sensitivity to missingness and was found to be relatively consistent. In this study, we only use individuals with complete therapy quality data and run analyses without multiple imputation.

The specific analysis of therapy quality presented in this paper was not pre‐planned in the original trial. We report the exploratory findings with the available data and describe the results with caution. We will quantify uncertainty in our reported estimates using confidence intervals and, for reference, Satterthwaite approximations were used for degrees of freedom.

## RESULTS

Table 1 presents the results from the moderator analysis using linear mixed effects models (LMM). The reference model is most like the original reported model from Jahoda et al. (2017) with the addition of baseline anxiety as an additional control. In this model, the difference in outcomes between the two interventions is not statistically significant (β = −1.64, [95% CI −6.60, 3.33]; *p* = .52) and only baseline GDS was statistically significant as a predictor of depression outcomes (β = .48, [95% CI .26, .70]; *p* < .001). Model 1 further included the TQM‐ID score, which does not have a statistically significant effect, with baseline GDS remaining statistically significant as a predictor.

**TABLE 1:** Linear mixed‐effects regression models for the baseline and three further models, 169 participants in each analysis.

Predictors	Reference model		Model 1 (including treatment quality)		Model 2 (including treatment quality and interaction with treatment)		Model 3 (including treatment quality, interaction with treatment and session in which quality was assessed)	
	Estimates	CI	Estimates	CI	Estimates	CI	Estimates	CI
(Intercept)	3.08	−1.77 to 7.92	2.98	−1.93 to 7.90	3.34	−1.55 to 8.24	2.96	−2.30 to 8.22
Base GDS	.48***	.26 to .70	.48***	.26 to .70	.47***	.25 to .69	.47***	.26 to .69
Baseline anxiety (GAS)	.11	−.05 to .26	.11	−.05 to .27	.11	−.05 to .26	.11	−.05 to .27
Time	−.85	−2.67 to .97	−.85	−2.67 to .97	−.84	−2.66 to .97	−.84	−2.66 to .97
Intervention (Beat It): Behavioural Activation	−1.64	−6.60 to 3.33	−1.58	−6.58 to 3.43	−1.57	−6.57 to 3.42	−1.54	−6.56 to 3.47
Centre: centre 2	.60	−2.65 to 3.85	.56	−2.74 to 3.86	.93	−2.37 to 4.23	1.01	−2.34 to 4.36
Centre: centre 3	−1.42	−5.46 to 2.62	−1.48	−5.60 to 2.63	−1.32	−5.41 to 2.77	−1.53	−5.79 to 2.73
Time × Behavioural Activation	.33	−2.33 to 2.99	.33	−2.33 to 2.99	.32	−2.34 to 2.99	.33	−2.34 to 2.99
TQM‐ID c			.04	−.29 to .37	−.27	−.72 to .17	−.28	−.73 to .17
Behavioural Activation × TQM‐ID_c					.62*	.01 to 1.24	.64*	.02 to 1.27
TQM‐ID session: TQM‐ID session 1							.51	−2.11 to 3.13
**Random effects**								
*σ* ^2^	18.77		18.78		18.81		18.81	
*τ*_00_	16.99_id: therapist_		17.15_id: therapist_		15.45_id: therapist_		15.58_id: therapist_	
	6.85_therapist_		7.15_therapist_		7.85_therapist_		8.14_therapist_	
ICC	.56		.56		.55		.56	
*N*	87_id_		87_id_		87_id_		87_id_	
	50_therapist_		50_therapist_		50_therapist_		50_therapist_	
**Model Fit Indices**								
AIC	1087.86		1091.47		1089.90		1089.43	
BIC	1122.28		1129.03		1130.59		1133.25	
*R* ^2^ conditional	.69		.70		.70		.70	
*R* ^2^ marginal	.31		.31		.33		.33	
ICC	.56		.56		.55		.55	
RMSE	3.42		3.42		3.41		4.40	
Sigma	4.332854		4.332352		4.326469		4.327238	
Observations	169		169		169		169	

* *p* < .05.

*** *p* < .001.

Model 2 included the TQM‐ID score and the interaction of TQM‐ID score and intervention. Here, the difference in interventions remained non‐significant but the TQM‐ID and intervention interaction was statistically significant (β = .62, [95% CI .01, 1.24]; *p* = .04); the TQM‐ID main effect itself remained not statistically significant and the baseline GDS remained a statistically significant predictor (β = .47, [95% CI .25, .69]; *p* < .001). Model 3 included an additional indicator of TQM‐ID session timing as a binary indicator of whether the therapy quality assessment was taken during an earlier or later intervention session; the effect of which was also not significant.

Table 1 shows the model fit indices for the nested LMM models (Reference, Model 1, Model 2 and Model 3); these indicate improved fit with lower values but showed only minor differences between the models and very minor improvement with inclusion of the additional covariates. We take forward Model 2 for further description as this result showed the most parsimonious model with interaction included, despite relatively minor differences in fit indices.

To examine the nature of the interaction, the TQM‐ID scores were plotted against the predicted GDS‐LD scores, separately, for 4‐month and 12‐month follow‐up. At lower levels of therapy quality, there is a clear difference between the GDS outcomes; GSH outcome scores are comparatively higher and BA scores are lower, as TQM‐ID score increases GSH scores reduce while BA scores improve, at higher levels of TQM‐ID score both interventions produce similar outcomes.

## DISCUSSION

This study has reported the moderating impact of a therapy quality measure for structured, time‐limited psychological therapies for people with intellectual disabilities based on data from a large‐scale RCT (Jahoda et al., 2017). The TQM‐ID was developed specifically for the purpose of this study but is based on well‐established measures and the consistency of therapy quality data collection in the study was carefully managed (Jahoda et al., 2018).

Moderator analysis of the primary outcome measure (GDS‐LD) using linear mixed effects models was carried out using four models, initially using key moderating variables, and then adding in a simple effect for TQM‐ID score, an interaction effect of TQM‐ID score with therapy type and the session in which the measure was taken. For each model, baseline depression score was a significant predictor, TQM‐ID score alone and session timing for the therapy quality measure were not significant predictors but the interaction of TQM‐ID score and treatment type was a significant predictor. The interaction graph shows that at lower levels of TQM‐ID score, there is a clear difference between the outcomes; for guided self‐help, outcome scores are comparatively higher and for behavioural activation, scores are lower, as TQM‐ID score increases guided self‐help scores reduce while behavioural activation scores improve, and at higher levels of TQM‐ID, scores both interventions produce similar outcomes. It should be noted that the opportunity to examine the interaction of therapy quality and treatment is a unique aspect of the design of this study as funders wished to see an ‘active’ rather than a ‘treatment as usual’ control and thus fidelity and outcome data were available for both arms. Similar interactions have not been tested in other studies.

The pattern of relationship of therapy quality and outcome for behavioural activation shows outcome improving as therapy quality increases. For the guided self‐help intervention, the relationship initially seems contradictory (although the association of components of therapy quality and outcome have not always been found to be consistent or linear, Branson et al., 2015). However, guided self‐help is a much simpler intervention where the ‘therapist’ is sometimes described as a ‘coach’ (Delgadillo, 2018) and the activities recorded in the TQM‐ID (e.g., more careful goal setting, close management of communication and developing and maintaining engagement of participants) might be indicative of GSH therapy where there are challenges. Competent delivery of GSH, where the aim was to help people with intellectual disabilities and their supporters to take control of the materials, may require a different therapeutic approach from more complex interventions. There are no other similar studies comparing therapy quality within RCTs, although studies have shown different short and long‐term outcomes for guided self‐help and face‐to‐face CBT (Salomonsson et al., 2018).

These results are novel and suggest that detailed analysis of therapy process in therapy with people with intellectual disabilities should be considered in future intervention research and that measures of therapy quality may need to be specific to the type of therapy used. The scale developed here was developed primarily to measure quality in the more complex and flexible approach of behavioural activation, the statistically significance for the interaction of TQM‐ID and intervention is very close to the alpha = .05 cut‐off and the analysis of fidelity presented in this paper was not pre‐planned in the original trial and so the study was not powered *a priori* to examine interactions of this type, thus the results should be interpreted with caution.

## AUTHOR CONTRIBUTIONS

**Dave Dagnan:** Conceptualization; formal analysis; methodology; funding acquisition; project administration; writing – review and editing; writing – original draft; investigation. **P. Thompson:** Conceptualization; data curation; formal analysis; writing – original draft; writing – review and editing. **R. P. Hastings:** Conceptualization; methodology; investigation; formal analysis; funding acquisition; project administration; writing – original draft; writing – review and editing. **C. Hatton:** Conceptualization; investigation; funding acquisition; methodology; project administration; writing – original draft. **C. Melville:** Conceptualization; methodology; investigation; funding acquisition; writing – review and editing. **S.‐A. Cooper:** Conceptualization; methodology; writing – review and editing; investigation. **N. McMeekin:** Methodology; funding acquisition; writing – review and editing. **L. Fulton:** Investigation; writing – review and editing; methodology. **R. S. P. Jones:** Conceptualization; methodology; investigation; funding acquisition; project administration; writing – review and editing. **A. McConnachie:** Conceptualization; data curation; formal analysis; funding acquisition; writing – review and editing. **A. Jahoda:** Conceptualization; methodology; investigation; funding acquisition; project administration; writing – original draft; writing – review and editing.

## CONFLICT OF INTEREST STATEMENT

None.

## Data Availability

The dataset for the analysis described in this paper will be made available on acceptance of the paper.
